# Supplementary figures and images for: Generative modeling for RNA splicing prediction and design
Source: eLife. 2026 May 29;14:RP106043. doi: 10.7554/eLife.106043 (PMC13221180; doi:10.7554/eLife.106043)

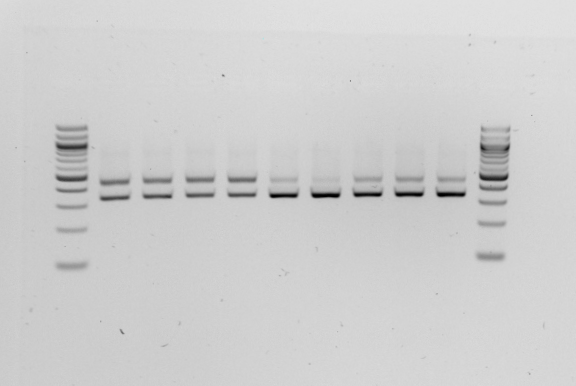

Supplement: Figure 5—source data 1. [file elife-106043-fig5-data1.zip › Figure5SourceData1/ACIN1_RT-PCR.tif]

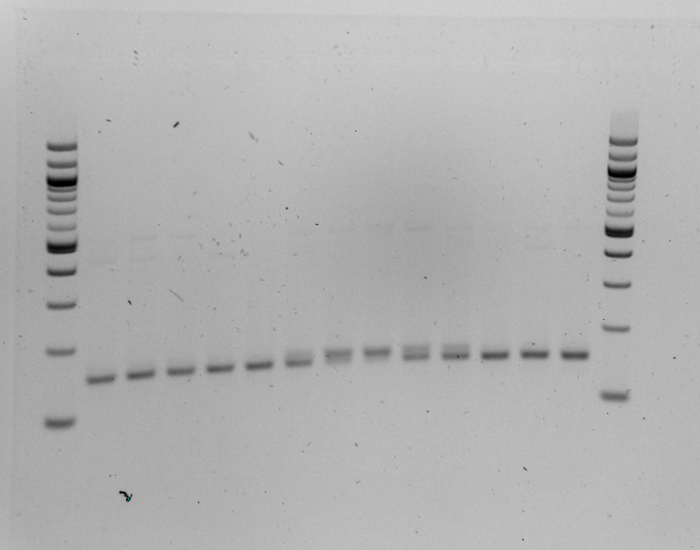

Supplement: Figure 5—source data 1. [file elife-106043-fig5-data1.zip › Figure5SourceData1/PTK2_RT-PCR.tif]

## *ACIN1* RT-PCR

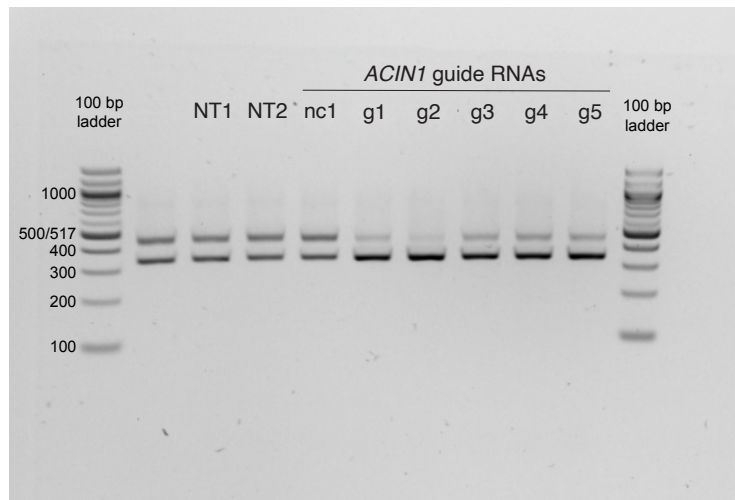

## *PTK2* RT-PCR

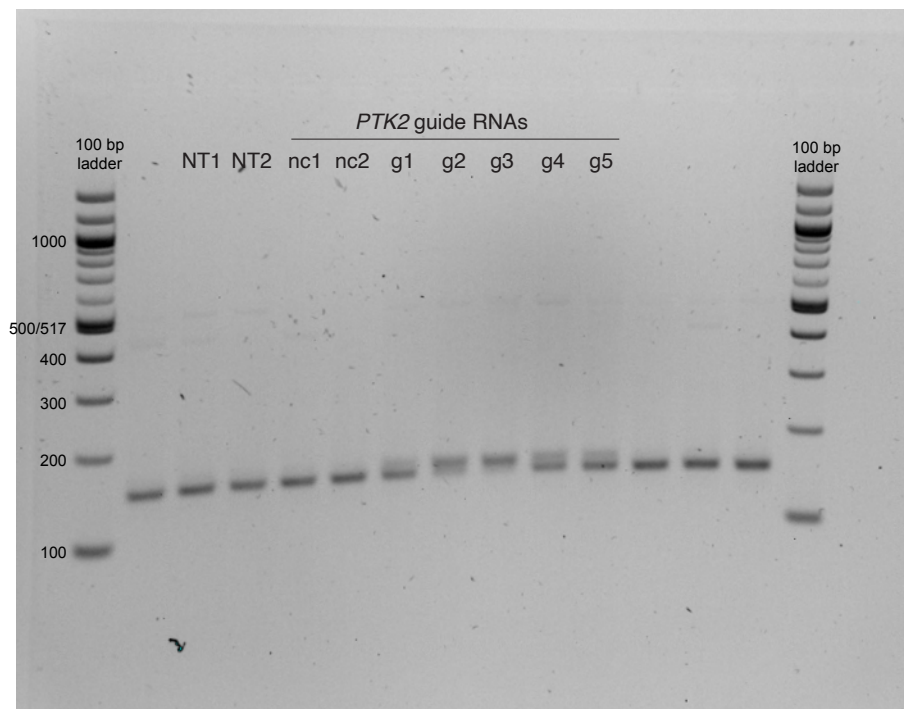

Supplement: Figure 5—source data 2. — Ladders are 100 bp DNA Ladder from NEB (N3231). [file elife-106043-fig5-data2.pdf]
